# Water-Cures in Peace and War: The Medical Bath in History

**Published:** 1915-06-05

**Authors:** 


### WATER-CURES IN PEACE AND WAR.

### The Medical Bath in History.

The first of a series of three Chadwick Lectures on this subject was delivered at the home of the Royal Society of Medicine on Thursday, May 27, by Dr. R. Fortescue Fox, F.R.Met. Soc. The chair was occupied by Sir James Crichton-Browne, F.R.S., one of the Chadwick Trustees, who, in his introductory remarks, said that there were now large numbers of soldiers who had been crippled by fractures and adhesions, and were suffering from rheumatism, fibrositis, arthritis, lumbago, sciatica, and a host of nerve lesions, as well as general neurasthenia; and baths and climatic conditions in conjunction with ordinary medicine and surgery were of infinite value in restoring them to health.

The lecturer said that, following the war, many troubles would remain after surgery, hospital treatment, and nursing had done what they could. He believed that there would be a still greater call for after-care in the case of large numbers of sufferers from this conflict. His first reference to baths was to those used by the Assyrians some 3,300 years ago: luxurious structures built of marble. They were everywhere associated with religious observance, and cleanliness was striven after not alone for its own sake, but because of its symbolic relation to the purity of the soul. This symbolism was seen in the case of the Pool of Bethesda, for it was only efficacious when troubled by the angel. The Greeks as a nation were attached to the cold bath; they bathed in rivers and other open streams, on account of the stimulus engendered; and it was a long time before they followed hot bathing. They then learned its power of dissipating fatigue. The douche bath was shown in paintings executed 600 B.C.; the water was poured over the body from gargoyle-like vessels. The Romans had various graded rooms attached to their bathing establishments, in which active and passive movements and massage were carried out; and anointing was a special function. At one time the city of Rome contained 170 public baths, and the acme in bathing luxury was reached by the great "Thermæ" of the emperors. One of the baths of Diocletian was a mile in circumference, and contained 16,000 seats of marble, while the walls were covered with costly mosaics. The vapour bath was less used by the Romans than by the Greeks. The Emperor Constantine was said to have bathed six to eight times a day, and this luxurious and frequent hot bathing the lecturer regarded as a factor in the national degeneration, as well as an indication of it.

Curiously, there were no traces in Constantinople of the Roman baths which once existed there, but throughout the present Turkish Empire there was scarcely a town of importance which had not its public bath. At certain periods the general use of the hot bath became a factor in the dissemination of such diseases as leprosy and syphilis. The sixteenth century witnessed a real advance in the scientific use of water treatment; but there was still no research into the actions and uses of waters, and watering-places remained centres of amusement. At the present day health resorts stood for a sane and scientific appeal to natural process; they provided slow remedies for slow processes, and were a valuable supplement to other forms of medical and surgical art in incipient and chronic diseases, for chronic disease seemed to be the deadlock to the natural process of cure.

The lecture was profusely illustrated by educational slides.

